# Research progress of cartilage lubrication and biomimetic cartilage lubrication materials

**DOI:** 10.3389/fbioe.2022.1012653

**Published:** 2022-10-04

**Authors:** Haoming An, Yubo Liu, Jiafeng Yi, Hongbin Xie, Chao Li, Xing Wang, Wei Chai

**Affiliations:** ^1^ Senior Department of Orthopedics, Fourth Medical Center of People’s Liberation Army General Hospital, Beijing, China; ^2^ School of Medicine, Nankai University, Tianjin, China; ^3^ National Clinical Research Center for Orthopaedics, Sports Medicine and Rehabilitation, Beijing, China; ^4^ Beijing National Laboratory for Molecular Sciences, Institute of Chemistry, Chinese Academy of Sciences, Beijing, China; ^5^ The Institute of Chemistry of the Chinese Academy of Sciences, University of Chinese Academy of Sciences, Beijing, China

**Keywords:** articular cartilage, cartilage lubrication, osteoarthritis, hydrogel, biomimetic materials

## Abstract

Human joints move thousands of times a day. The articular cartilage plays a vital role in joints’ protection. If there is dysfunction in cartilage lubrication, cartilage cannot maintain its normal function. Eventually, the dysfunction may bring about osteoarthritis (OA). Extensive researches have shown that fluid film lubrication, boundary lubrication, and hydration lubrication are three discovered lubrication models at cartilage surface, and analyzing and simulating the mechanism of cartilage lubrication are fundamental to the treatment of OA. This essay concludes recent researches on the progress of cartilage lubrication and biomimetic cartilage, revealing the pathophysiology of cartilage lubrication and updating bio-inspired cartilage lubrication applications.

## 1 Introduction

With the primary function to ensure normal joint movements, a joint’s components consist of articular cartilage, ligaments, synovial fluid, joint cavity, and synovial membrane. Articular cartilage locates at the end of the joint which is a layer of connective tissue about 1–4 mm thick (Naumann et al., 2002; Burstein et al., 2009) without blood vessels, nerves, and lymphatic vessels, whose nutrition comes from synovial fluid and subchondral bone (Archer et al., 1990; Sophia Fox et al., 2009). Articular cartilage conducts and distributes loads during joint movements, and normal human articular cartilage has the most efficient lubricating surfaces known in nature, such as the knee and hip joints. In daily life, for example, walking on flat ground, the load on articular cartilage is about 5 MPa (49.3 atm) (Marzo and Gurske-DePerio, 2009; Venäläinen et al., 2016). For the surface of articular cartilage, the coefficient of friction (CoF) is exceedingly low, which can reach 0.001 under physiological pressure, which provides the necessary guarantee for normal joint movements. For common diseases such as osteoarthritis (OA), the main cause of which is degenerative lesions of the articular cartilage, secondary to other clinical symptoms, the prevention of such diseases, i.e., treatment, is centered on restoring or improving the lubricity of the articular cartilage surface. This report reviews several known mechanisms of joint lubrication, as well as recent advances in hydration lubrication. In addition, recent advances in biomimetic cartilage materials and their tribology theories are reviewed.

## 2 Cartilage structure and composition

The main compositions of articular cartilage are chondrocytes and the surrounding extracellular matrix. Chondrocytes is the single kind of cells in articular cartilage that is derived from mesenchymal stem cells, whose function is maintaining the cartilage matrix. The extracellular matrix comprises water, aggrecans, collagen (mainly collagen II), and small amounts of phospholipids, other proteins, glycoproteins, and lipids (Sophia Fox et al., 2009). Several macromolecules are synthesized and maintained by chondrocytes, of which collagen II is the main fiber in the extracellular matrix, accounting for about 55%–75% of the dry weight of cartilage.

**FIGURE 1:**
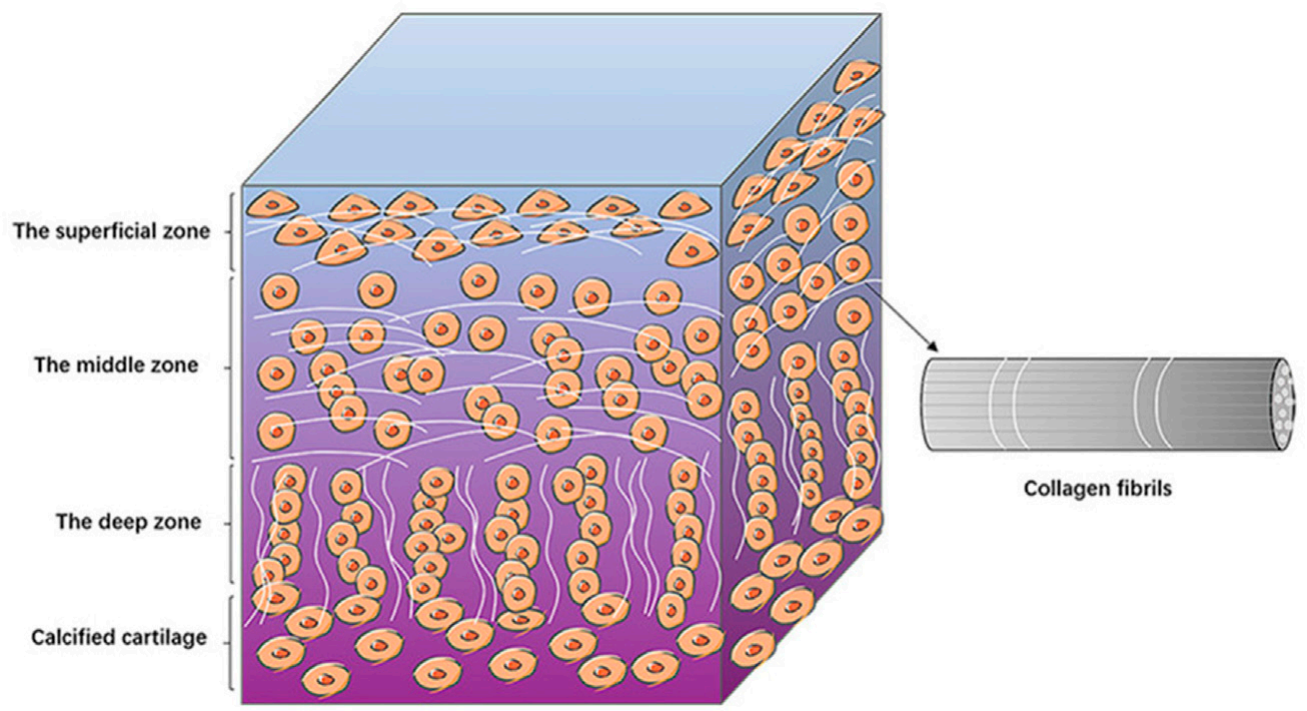
The structure of articular cartilage. The diagram shows the basic structures of the surface, middle and deep zones of the articular cartilage. Chondrocytes are arranged differently in different zones. The collagen fibers are lined up with chondrocytes (Jiang et al., 2022). Copyright 2022, Am J Transl Res.

Articular cartilage has no blood vessels or lymphatic vessels and is not innervated; and it relies on the diffusion of synovial cells for nutrients and metabolism. It is difficult to obtain nutrients in this way, so the cartilage tissue has a limited thickness and is less capable of self-repair. Depending on the structure and components, articular cartilage can be divided into three areas, the surface zone, the middle zone and the deep zone (Żylińska et al., 2021). In surface zone, the chondrocytes have a flattened morphology and their long axis is parallel to the cartilage surface. The collagen fibers (mainly collagen II fibers) are arranged vertically, so that they can withstand the shear forces during joint movements (Mow et al., 1984). In middle zone, the morphology of the chondrocytes is round, with a reduced number compared to the surface zone, and the collagen fibers are sparsely distributed, mainly to share and transmit the load. The chondrocytes in deep zone are arranged in bunches, and the fibers are parallel to each other, perpendicular to the joint surface from an overall perspective, making it possible to enhance cartilages’ compression resistance while also helping to anchor the cartilage to the subchondral bone surface (Mow et al., 1984). Because of this orderly layering of tissue fiber’s structure, cartilage has excellent mechanical properties (Figure 1).

The pathophysiology of cartilage leads to the need for lubrication. Human joints are subjected to thousands of movements per day, and the articular cartilage is subjected to different types of stresses and shear stresses. Under static stress or excessive dynamic stress, the anabolic rate of chondrocytes decreases or even apoptosis occurs, whereas under appropriate dynamic stress and dynamic shear stress, the anabolic rate of chondrocytes increases (Grodzinsky et al., 2000; Fitzgerald et al., 2006; Anderson and Johnstone, 2017). At the same time, cartilage aging, cartilage trauma, and changes in synovial fluid composition causes a decrease in the lubricating properties of articular cartilage and an increase in interarticular cartilage friction. Jay et al. (1992) found through a study of knockout mice that cartilage in the absence of effective lubrication resulted in destructive deformation of the cartilage surface, which in turn led to apoptosis of chondrocytes (Waller et al., 2013). Bonnevie et al., 2018 found that high friction caused the high shear strain on the articular cartilage surface, which was related to chondrocyte mitochondrial dysfunction, apoptosis, and cell death (Bonnevie et al., 2018). In recent studies, the heterogeneity of chondrocytes is gradually being discovered. Sebastian et al. (2021) profiled articular cartilage from healthy and injured mouse knee joints at a single-cell resolution and identified nine chondrocyte subtypes with distinct molecular profiles and injury-induced early molecular changes in these chondrocytes. The discovery of chondrocytes heterogeneity is inspiring for the study of chondrocyte lubrication mechanisms and the restoration of cartilage lubrication in patients with OA (Ji et al., 2019; Chou et al., 2020).

## 3 Models of lubrication at cartilage surfaces

The mechanisms of articular cartilage lubrication have been of interest and importance, and many researchers have devoted themselves to the exploration of the mechanisms of lubrication. Since the 1860s, many models of cartilage lubrication have been proposed. The first one was fluid film lubrication, in which researchers suggested that there was a low-friction film between the articular cartilage surfaces. As relative researches progressed further, many other lubrication models were proposed, such as squeeze-film mechanism, weeping lubrication, boundary lubrication, and hydration lubrication, etc. Among them, boundary lubrication and hydration lubrication are the focus of research today. Nowadays, more and more studies have shown that cartilage lubrication involves not only one mechanism, but multiple mechanisms are involved together, therefore, a detailed review of cartilage lubrication mechanisms is of necessity.

### 3.1 Fluid film lubrication

Fluid film lubrication refers to the exudation of tissue fluid to form a fluid film between joint surfaces when articular cartilage is compressed, and this film acts as a load-supporting and lubricating agent between joint surfaces (Smith et al., 2019). Back in the 16th century, Paracelsus discovered that the fluid in the joint cavity had an oil-like consistency and coined the term synovia to describe *synovial* fluid (Pagel, 1982). In the 1880s, research on hydrodynamic lubrication by Reynolds (1886) drove the study of cartilage lubrication mechanisms. In the 1930s, many theories of joint lubrication has begun to be proposed, including fluid film lubrication. In Macconaill (1932) discovered that the synovial membrane formed by the synovial membrane plays an important role in cartilage load-bearing and lubrication, and summarized an important theory about joint lubrication.

#### 3.1.1 Squeeze-film mechanism

However, the foundation of fluid film model is based on the hypothesis that the surface of articular cartilage is rigid. In the 1860s, the deformability of articular cartilage was considered. The lubricant viscosity increases under high pressure, and the two objects in contact undergo elastic deformation. Fluid film model is no longer applicable. The relative motion speed of the joint in bearing heavy load is generally low, or even zero, which is not beneficial for the establishment of thick lubrication film; while the relative motion speed is larger when the action load is small, or even zero, which is conducive to the formation of thick lubricating film. The change of lubricating film thickness during the movement of the joint takes time, so the thicker lubricating film established by the joint at high speed and light load will contribute to its lubrication at low speed and heavy load condition, which is the core of the squeeze-film mechanism. Unsworth et al. (1975) found that the resulting squeeze film bearing capacity is quite large. He deduced that joints were lubricated primarily by the squeeze-film at low speeds and heavy loads. He designed a test rig to conduct an experimental study on the lubrication mechanism of the joint. When there are heavy loads, the film’s thickness on the joint’s surface thins or even disappears, leaving the surface in a boundary lubrication state.

#### 3.1.2 Weeping lubrication and squeezing lubrication

Due to the porous and fluid-rich characteristics of articular cartilage, the fluid in the cartilage will penetrate the joint surface under load, thus lubricating the joint’s movement, a phenomenon known as weeping lubrication. The peculiarity of cartilage is also beneficial to the fluid film lubrication of the joint, as the cartilage acts as a fluid store when the joint is not under load, while when the joint is under pressure, the continuous outflow increases the thickness of the joint lubrication film. In extreme cases, the synovial fluid between the cartilages can be squeezed into a gel-like substance, leading to a notable increase in the load-bearing capacity of this fluid film, called squeezing lubrication.

### 3.2 Boundary lubrication

Since the pressure between articular cartilages’ surfaces can be as high as 100–200 atm (10.1–20.3 MPa), and under this high pressure, any fluid will be squeezed out, resulting in partial or total loss of the fluid film between the cartilage and local molecular or complete molecular contact between the cartilage. When there is only local molecular contact, both fluid film and boundary lubrication between the cartilage will be activated, while friction does not change regardless of the rate (Wright et al., 1973), which also suggests the formation of a hybrid lubrication mechanism. When the whole molecule is in contact, the main lubrication mechanism between the articular cartilage is the boundary lubrication mechanism.

**FIGURE 2:**
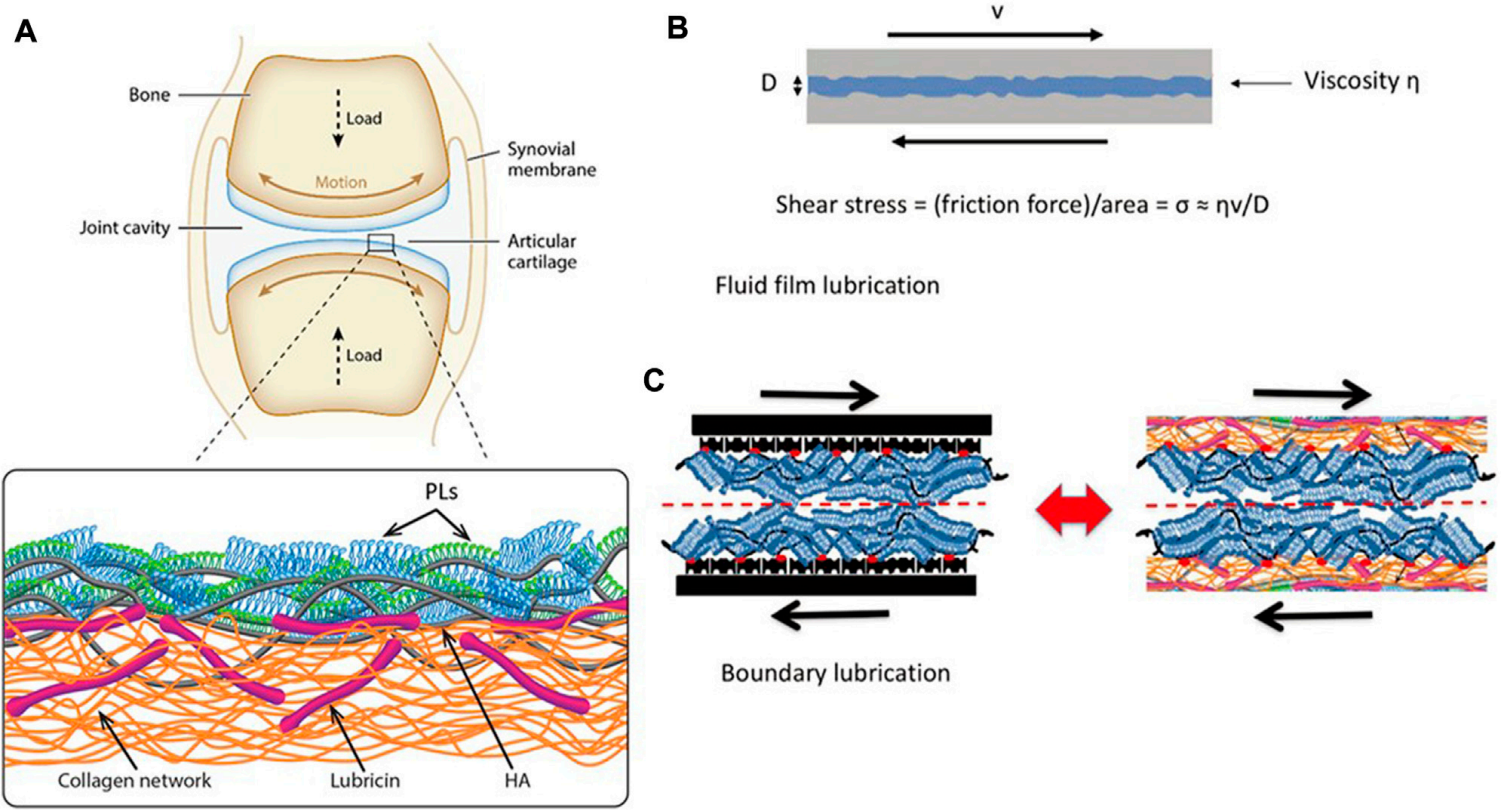
**(A)** Illustration of a synovial joint and its boundary layer consisting of linear HA (gray), mucinous glycoprotein lubricin (purple), and phospholipids (monolayer, green; bilayer, blue) on a collagen network (orange). **(B)** Illustration of a fluid-film lubrication model. When the joint moves, a fluid layer between cartilage surfaces separates the cartilage surfaces, reducing friction and protecting the articular cartilage. The shear stress σ_s_ may be written in the Newtonian form, σ_s_ = (shear rate) × (effective fluid viscosity). **(C)** In this boundary lubrication model, friction occurs between the interface of the articular cartilage surface, not in the substrates beneath it. The frictional dissipation in the boundary lubrication model is closely linked to the molecules in boundary layers (Lin and Klein, 2022). Copyright 2022, Acc Mater Res.

Boundary lubrication refers to forming a thin adhesive layer (1–10 nm) on the articular cartilage contact surface to maintain surface separation and prevent adhesion under load (Fan et al., 2012). Boundary friction depends on the property of the boundary layers in contact with each other, which is due to the fact that this kind of friction is caused by the sliding of the contact surfaces between the boundary layers, inspect of the substrate layer. Common boundary substances include hyaluronic acid (HA), aggrecans, lubricin, and phospholipids. In recent years, many experiments were conducted to determine the constituent of molecular boundary layers to understand the mechanisms of boundary lubrication, and the following section will focus on common boundary layer substances (Figure 2).

Hyaluronic acid (HA) is an acidic mucopolysaccharide, which was first isolated from the vitreous humor of bovine eyes by Meyer et al. (1983) at Columbia University in 1934. Hyaluronic acid plays an important role in the lubrication of joints due to its unique molecular structure and physicochemical properties. HA was initially considered to be the molecule that acts as a lubricant on the cartilage surface (Swann et al., 1974) until a subsequent experiment demonstrated that the enzymatic digestion of HA in synovial fluid resulted in little change in cartilage lubrication capacity (Radin et al., 1970). Nevertheless, HA is also frequently used as a viscous supplement for the relief of clinical symptoms in patients with OA. The viscosity of HA solutions and synovial fluid becomes similar to that of water when crossed over articular cartilage surfaces at corresponding shear rates (Krause et al., 2001). In contrast, some experiments directly measured the CoF of models with surfaces coated by HA in physiological pressure. The results demonstrated HA’s terrible capability of boundary lubrication, and therefore HA cannot be considered a significant lubricating molecule on the cartilage surface (Lin and Klein, 2021). It has been shown (Jahn et al., 2016) that HA attached with aggrecan molecules has superior capability of lubrication with a CoF value µ ≈ 0.01, but only if the pressure is within a few atmospheres; if the pressure exceeds this value, the µ value increases sharply. At the same time, HA promotes wear protection of the cartilage surface and protects it from damage even at pressures up to 200 atm (20.3 MPa).

Lubricin is an elongated mucinous glycoprotein about 200 nm long and 10 nm wide. Lubricin is encoded in humans by PRG4 and is present in synovial fluid and surface layer of cartilage. In 1968, Linn and Radin et al. demonstrated that the removal of HA from the surface of dog joints did not lead to deterioration of lubrication. Conversely, when the proteins on the joint surface are digested and broken down by trypsin, their CoF increases. They then hypothesized that a specific protein was necessary for lubricity and speculated that the function of this protein is to anchor HA to the cartilage and promote cartilage surface lubrication. Moreover, several subsequent studies directly confirmed this connection (Jay et al., 1992; Chang et al., 2009). Radin et al. (1970) suggested that the portion of synovial fluid that had HA removed contained a lubricant. Swann et al. (1981) identified this substance as a glycoprotein and named it lubricin. Early tests showed that solutions containing lubricin reduced friction by about twofold relative to salt solutions without lubricin or solutions containing HA (Radin et al., 1970). Many subsequent studies demonstrated increased friction and wear in the cartilaginous joints of rats deficient in lubricin, proving that lubricin is critical in cartilage lubrication. However, some directly measured experiments demonstrated that lubricin is not an effective lubricant, particularly in the high-stress environment of articular cartilage, and the exact lubrication mechanism of lubricin in articular cartilage surface lubrication remains uncertain (Hills, 2002).

Phosphatidylcholines (PCs), amphiphilic molecules consisting of two hydrophobic tails and a hydrophilic phosphatidylcholine group, are widely found in synovial fluid and cartilage and are also considered to be an essential part in boundary lubrication. In 1984, Hills and Butler (1984) proposed the surface-active phospholipids (SAPL), which are generally referred to as phosphatidylcholine with a diacyl tail and phosphorylcholine head group and are the main lipid component of synovial fluid and articular cartilage surfaces (Jahn et al., 2016). They suggested that since the cartilage surface matrix is negatively charged, the PC attached to it should have a phosphorylcholine head attached to the cartilage matrix, exposing the hydrophobic acyl tail. The two hydrophobic tails slide against each other in the relative sliding plane to achieve lubrication and reduce wear between cartilage surfaces. However, this mechanism of frictional dissipation is generated by the breakage and reproduction of relatively weak van der Waals forces between the contact surfaces of the hydrophobic tails, and the CoF of it is about 0.05–0.1 (Lin and Klein, 2021), which is much higher than that between healthy human articular cartilage, indicating the model may be incorrect. Hills also found that when a mixture of HA and 1,2-dipalmitoyl-sn-glycero-3-phos-phocholine (DPPC) in the form of multilamellar vesicles (MLVs) was added to saline, the CoF when sliding between quartz surfaces was relatively low, about 0.004 (at a pressure of 3 atm, 0.3 MPa, a pressure more than 50 times lower than the maximum inter-articular pressure) (Jahn et al., 2016). In contrast, when only DPPC was added, the lubrication was better, with µ values of about 0.01–0.1. Overall, there are no studies that can accurately elucidate the mechanism of PC in boundary lubrication so far.

### 3.3 Hydration lubrication

The biological role of water molecules stems mainly from the dipole nature of their molecules and their hydrogen bonding ability, and we are interested in how they play the role in lubrication, i.e., how the water molecules in them regulate the frictional dissipation between two surfaces when two interfaces slide against each other. A study 20 years ago showed that water has long-lasting fluidity, and its viscosity is similar to regular water when confined to a film of molecular layer thickness (Raviv et al., 2001); whereas most organic solvents and oils change their viscosity and behave as solids when confined to a film of this similar thickness (Klein and Kumacheva, 1995). A study by Lin and Klein (2021) also explains the reason for this different state at similar film thicknesses but presents a different state: the confining surface attracts the confining liquid, thus making it dense. However, since water is denser than ice, densification causes water to be less likely to solidify (Lin and Klein, 2021).

Hydration lubrication is achieved by forming a sub-nano hydrated shell that significantly reduces frictional dissipation during gliding. This mechanism has been proved in other systems ranging from compressed charged surfaces sliding over captured hydrated counterions to more complex boundary lubrication systems that include surfaces composed of polyzwitterionic brushes, phosphatidylcholine (PC), hydrated surfactants, biomacromolecules, and bionic polymers boundary layers. In particular, phosphatidylcholine groups provide efficient lubrication (µ ≤ 10^−4^) either as monomers on polymer brushes or as surface-attached PC lipids or lipid bilayers (Lin and Klein, 2021). When the PC lipid components slide against each other, lubrication between them occurs at the interface between the hydrated phosphorylcholine headgroup layers, through the PC lipids in the cartilage boundary layer that hydrated lubrication reduces friction. In a recent surface force balance (SFB) experiment (Lin and Klein, 2021), studying the friction of boundary layer phospholipids extracted from human and bovine synovial fluid and cartilage showed that at high pressures (≥100 atm, 10.1 MPa), they still provided extremely effective lubrication (μ < 0.001). In hydration lubrication, a very low viscosity hydrated layer exists between the contact surfaces even under high pressure. They are immobilized by PC, and the ability of the PC bilayer to be very resistant to high pressure is due to the strong interaction between the lipid tails.

It has been shown that dissipation occurs in different ways under different pressures and shear rates, with cross-dissipation at high loading states mainly through the activation of the energy barrier caused by the localization of counterions on the confining surface. However, at low loading, it is the dissipation caused by the shear hydration shell itself (Ma et al., 2015a).

### 3.4 Synergies in cartilage lubrication

After the above explanation of the three cartilage lubrication mechanisms, we can see that hyaluronic acid, lubricin, and phospholipids molecules are commonly found in synovial fluid and cartilage, and all play an essential role in cartilage lubrication. However, each of them alone cannot provide nearly joint-like effective lubrication under the high pressure that the joint is subjected to, and many studies have found that multiple molecules in them work in concert with each other. Many studies have also found that multiple molecules in them work in concert to produce their effective lubrication.

Earlier experiments showed that the higher friction (μ ≈ 0.3) between mica surfaces carrying HA alone might due to the weaker hydration between HA monomers. This weaker hydration reduces the efficiency of hydrated lubrication under high pressure, resulting in higher energy dissipation (Seror et al., 2015). Seror et al. (2015) conducted an experiment in which HA was attached to a cartilage-like surface and found that HA bound to phosphatidylcholine (PC) among synovial joints to form strong boundary layers that provided deficient friction properties similar to cartilage (μ ≈ 0.001) even at the highest physiological pressures. In an experiment by constructing a sliding model within the tendon sheath of the synovial ends, Lin et al. (2019) found that liposomes with HA alone or PC liposomes reduced the tendon sheath CoF by approximately 40%–50% relative to the saline control. However, their combined use reduced the CoF to a 5-fold, suggesting that sliding biological tissues can be effectively lubricated by these HA and PC liposomes combined with a boundary lubrication layer. Zhu et al. (2017) performed boundary lubrication between surfaces with hyaluronic acid attached. In monolayer vesicles, these surfaces were incorporated with different phosphatidylcholine lipids (including HSPC, DMPC, and POPC). The results showed that the HA-HSPC complex lubrication was very effective, with a CoF value of about 0.001 at physiological pressure (*p* ≈ 150 atm, 15.2 MPa), indicating the synergistic effect of HA and PC lipids to achieve efficient lubrication.

Lubricin is an essential boundary lubricant, and many studies have focused on synergistic lubrication regarding its role in cartilage lubrication. Israelachvili et al. found that lubricin prevented wear and reduced friction by surface grafting lubricin-infused hyaluronic acid and hyaluronic acid. Raj et al. (2017) anchored cartilage oligomeric matrix protein (COMP) anchored on a moderately hydrophobic polymethyl methacrylate (PMMA) surface and found that the lubricating ability of lubricin was enhanced.

## 4 Bio-inspired cartilage lubrication applications

### 4.1 Hydrogel

Hydrogels, one of the most suitable scaffold biomaterials for tissue engineering and regenerative medicine, is a class of extremely hydrophilic, three-dimensional network structured gels that swell rapidly in water with characteristics such as high-water absorption, biodegradation, adjustable porosity, and biocompatibility like that of the natural extracellular matrix (ECM) (Wang et al., 2020). It is widely used in biomedical devices and regenerative medicine. However, conventional hydrogels have a single energy dissipation mechanism, poor load-bearing, and resistance to loss. Their performance cannot be compared with that of natural cartilage, so the improvement of its mechanical properties is the only way to better apply hydrogels to cartilage lubrication. Researchers have developed many high-strength and efficient hydrogels, such as double network (DN) hydrogels, nanocomposite hydrogels, and ionic cross-linked hydrogels.

#### 4.1.1 High performance hydrogel cartilage material

DN hydrogels are a class of stiff hydrogels consisting of a relatively rigid polyelectrolyte as the primary network and a neutral polymer with low cross-linkage as the secondary network (Lin and Klein, 2021), first polymerized by Gong et al. DN hydrogels improve strength by changing the topology of their network itself and increasing the physical cross-linking points between the polymer chain ends. Means et al. (2019) investigated the mechanical properties of DN hydrogels on porcine cartilage, and they found that the strength, modulus, and hydration of DN hydrogels were comparable to those of healthy cartilage. The CoF was reduced by 50% compared to conventional single-network hydrogels. However, the energy dissipation of DN hydrogels depends almost entirely on the first mesh breakage, and in practice, the mechanical properties dominated by the second mesh will be significantly reduced after the first mesh breakage. The CoF of DN hydrogels is also relatively large, μ ≈ 0.1. To solve this problem, Kaneko et al. (2010) designed the triple-network hydrogels by adding a third, negatively charged, weakly cross-linked network to the structure of the DN hydrogels, as well as the same polymer chain as the main network in the original DN hydrogels. The CoF of the triple-network hydrogels are significantly lower than the DN hydrogels at the same motion speed. Milner et al. (2018) incorporated a polymer of the biomimetic boundary lubricant poly(2-methacryloyloxyethyl phosphorylcholine) (PMPC) into the DN hydrogels to form a PMPC triple network hydrogels with excellent lubrication performance, which had half the friction of the DN hydrogels at the same motion speed. It had a yield stress of 26 MPa (256.6 atm, 26 MPa), which was higher than that of the natural human knee joint, with a peak stress higher too. This approach can also be used in biological tissues to improve wear resistance.

Since cartilage is a laminar porous structure with many glycosylated hydrated molecular chains distributed on its surface, the researchers have designed a new bilayer hydrogel that can penetrate the surface with hydrated macromolecular chains and synovial fluid to provide lubrication when the cartilage is under pressure. The researchers constructed a new integrated bilayer hydrogel by blending polyvinyl alcohol (PVA) and β-tricalcium phosphate (β-TCP), which has significantly improved mechanical properties with cartilage-like lubrication (μ ≈ 0.05) and high biocompatibility. This novel bilayer hydrogel exhibited powerful biological functions to promote cartilage regeneration after co-culture with chondrocytes and synovial interstitial mesenchymal stem cells (Yao et al., 2017).

Ionic cross-linked hydrogels are hydrogels in which metal-ligand coordination bonds are cross-linked with ionic bonds formed by anions and cations in a hydrogel network, in which the coordination and ionic bonds can dissociate and join rapidly and reversibly, thus dissipating energy when stressed. These hydrogels have self-healing properties and fatigue resistance, but their poor mechanical strength also limits their application (Yang et al., 2018). Based on this principle, Sun et al. (2012) constructed a mechanically sound double-network hydrogel with extremely high toughness, high tensile rate (≈2,000%) and insensitivity to crack defects.

Many hydrogels are designed to be porous-filled. Based on this, Mu et al. (2020) soaked macroporous hydrogels in an aqueous solution containing movable polymer chains and placed them on a glass to slide, with an order of magnitude lower friction than hydrogels without polymer filling. Lin et al. (2020) added PC lipids in the form of multilayer vesicles to form hydrogels through polymerization and cross-linking. These lipids form a lipid micro-reservoir within the hydrogel, and the lipids in the micro-reservoir can be continuously released during surface attrition to remodel the cartilage surface to form a self-lubricating hydrogel. At the same time, this hydrogel exhibits consistently low friction and very low wear compared to the non-lipid hydrogel. The percentage of lipid volume added is about 1% or even lower, so there is almost no effect on the mechanical properties of the hydrogel. This dual cross-linking (DC) network hydrogel has a customized structure and excellent mechanical properties to meet a wide variety of clinical needs (Dou et al., 2020).

In addition to the ionic cross-linked hydrogels, Zhang et al. (2022) prepared a semi-interpenetrating network composite gel (CG) by incorporating short chain chitosan (CS) into a covalent tetra-armed poly(ethylene glycol) network. This new structure has excellent physicochemical properties and the ability to carry drugs.

#### 4.1.2 Surface lubrication modification of hydrogel cartilage material

There are many brush-like hydration molecular chains on the surface of natural articular cartilage, and these molecular chains can enhance the hydration of the laminar porous structure on the cartilage surface, thus allowing the joint to achieve low friction even under high load. So many researchers started investigating the grafting of some hydrophilic polymer brushes onto the hydrogel surface to improve interfacial hydration and reduce friction. Initially, the polymers were immobilized by physical grafting, but the grafting rate was influenced by the spatial site resistance, resulting in a low polymer density on the surface of the grafted hydrogel, which could not produce sufficient hydration. At the same time, the interfacial bonding of physisorption is relatively weak, and the polymer brushes on the surface are highly susceptible to shear, leading to lubrication failure. Thus, researchers have gradually adopted chemical grafting methods with higher grafting rates and stronger binding capabilities. Most current methods using surface-initiated radical polymerization (SI-ATRP) treat the substrate, but the conventional SI-ATRP polymer brushes are only anchored to the surface. They do not attach to the deeper layers, resulting in high susceptibility to wear under high loading conditions at the joint, affecting cartilage function. Researchers have therefore started to use chemical interpenetration synthesis in polymer networks. Rong et al. (2020) grafted very thick (≈10 μm) polymer brushes on the surface of a hard hydrogel that was able to maintain very low friction (order 0.01) under high loading. Du et al. (2016) embedded initiators in the polymer substrate to graft polymer layers to enable the grafted polymer layer to re-initiate polymerization after wear.

**FIGURE 3:**
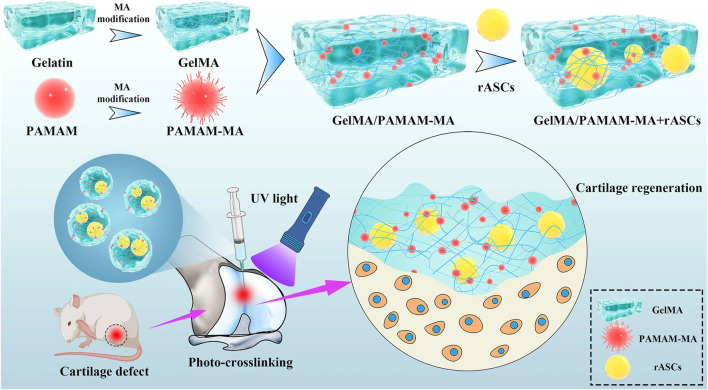
Schematic diagram of the injectable stem cell-laden photocrosslinked GelMA/PAMAM-MA hydrogel for cartilage regeneration (Liu et al., 2022) Copyright 2022, Stem Cell Research & Therapy.

Liu et al. (2020) used SSI-ATRP chemistry to graft polymer brushes onto initiator-embedded temperature-sensitive hydrogel substrates. The bottom hydrogel substrate could automatically modulate the load-carrying capacity based on temperature changes, preparing high-strength hydrogels with temperature-regulated lubrication properties. In addition, Qu et al. (2020) developed a layered hydrogel material consisting of a soft and porous surface layer (which can store lubricant) and a solid bottom layer by using an alkali-induced network dissociation strategy, where the lubricant in the surface layer can be released during sliding, achieving an extremely low CoF on the surface (μ ≈ 0.009 for a 3 N load and μ ≈ 0.035 for a 20 N load). This reliance on the synergistic effect of surface lubrication plus bottom loading achieves excellent lubrication and wear resistance. Liu et al. (2022) constructed the photocrosslinked gelatin methacrylate (GelMA) hydrogel which has higher cell affinity and plasticity. This kind of hydrogel exhibits more uniform bodies, better stability, and superior mechanical properties (Figure 3).

Yue et al. (2022) prepared a brush-like chitosan-g-PMPC copolymer by one-step grafting in the aqueous medium. This brush-like copolymer has efficient lubrication (CoF μ ≤ 0.01 on artificial articular cartilage substrates such as Ti6Al4V alloy and degraded bovine articular cartilage surfaces) and good biocompatibility due to the hydration properties of PMPC side chains, copolymer interface adsorption, and hydrodynamics of the copolymer. The polymer has excellent lubricating properties, while *in vivo* experiments have demonstrated its role in reducing the symptoms of arthritis swelling, promising its use for arthritis treatment and artificial joint lubrication. Zhao et al. (2022) combined the load-bearing phase of the 3D elastic scaffold hydrogel matrix with the surface lubrication layer of a polyelectrolyte brush, and the hydrophilic polymer brush was chemically grafted onto the 3D elastic scaffold-hydrogel subsurface. Since the 3D elastic scaffold can disperse stress and store elastic energy applied to the sample, energy dissipation does not need to be accomplished using sacrificial networks or broken bonds. This non-dissipative mechanism combined with interfacial lubrication gives the complex excellent wear reduction and wear resistance under dynamic shear, and the average CoF of the lubricated phase of the composite measured using conventional reciprocating tribological instruments is about 0.1, hence its good use in the field of joint bionic materials.

Huang et al. (2021) fabricated a soft hydrogel-elastomer hybrid surface by interpenetrate the hydrogel into the polymeric elastomer substrate as a transitional and bonding zone. The material exhibited a low CoF and wear-resistance under a high load. Researchers (Zhao et al., 2020) used 2-methacryloyloxyethyl phosphorylcholine (MPC) and N-(3,4-dihydroxyphenethyl)- methacrylamide (DMA) to synthesize system P (DMA-co-MPC) through a series of synthesis, which showed good performance in the scavenging of oxidation groups and lubricating properties, mainly due to the antioxidant function of the hydroquinone portion of DMA and the hydrated lubricating effect of the zwitterionic phosphocholine group in MPC. Lei et al. (2022a) fabricated a hyaluronic acid-based rapamycin liposome (RAPA@Lipo@HMs), which improved the joint lubrication by using a smooth rolling mechanism and continuous release of liposomes from the outer surface due to wear to form a self-regenerating hydration layer. At the same time, RAPA@Lipo@HMs reduces joint wear and delays the progression of Osteoarthritis, providing effective joint lubrication. The initial CoF μ value of this liposome was 0.04, and it was able to directly reduce the CoF μ to 0.03 in a brief period of time in the 3,600 s friction test and maintained this low value in subsequent tests. Since the lattice structure cannot be changed once the conventional hydrogel is formed, once the boundary layer is damaged due to shear, the lubrication provided by the hydrogel becomes unstable as the internal liposomal microcarriers cannot be exposed until the outer surface wears off. Inspired by this, Lei et al. (2022b) added liposomes containing celecoxib (CLX) to a dynamic covalently bonded hyaluronic acid hydrogel (CLX@Lipo@HA-gel) to form a shear-responsive boundary-lubricated drug-loaded hydrogel, which provides stable lubrication due to a dynamic cross-linked network that allows the hydrogel to reorganize in response to shear. In contrast, the hydrogel matrix allows the internal liposome microcarriers to accumulate on the sliding surface. The initial μ-value of CLX@Lipo@HA-gel measured in the experiment was about 0.031 (similar to the initial value of HA-gel), and the μ-value decreased continuously with time to form stable lubrication. At the same time, the ordinary HA-gel had a poor lubrication effect due to a sharp decrease in μ-value followed by an increase in μ-value after a short period of water loss. Therefore, CLX@Lipo@HA-gel hydrogels have promising applications in diseases such as OA due to their excellent shear properties and as a drug carrier. The researchers successfully constructed hydration-enhanced lubricating nanofibers which is about 65% lower than pure polycaprolactone (PCL) nanofibers and has an excellent anti-wear and strong anti-adhesion ability by one-step *in situ* grafting of a copolymer synthesized from dopamine methacrylamide (DMA) and 2-methacryloyloxyethyl phosphorylcholine (MPC) onto electrospun PCL nanofibers (Cheng et al., 2020).

Yang et al. (2020), based on the adhesion properties of ball bearings and mussels, fabricated a highly monodisperse photocrosslinked GelMA microspheres (denoted as MGS) with controlled particle size. And then, they spontaneously modified the microspheres with dopamine methacrylamide-to-sulfobetaine methacrylate (DMA-SBMA) copolymer by a one-step bionic grafting method. The microspheres have enhanced lubrication with more effective drug loading and release capacity while reducing the wear between sliding cartilage surfaces. The CoF of MGS@DMA-SBMA in reciprocal friction type experiments decreased approximately 25% compared to MGSs (μ = 0.032). In contrast, the CoF of the former did not decrease significantly with increasing load, indicating its stable structure and excellent lubricating properties. In addition, they successfully prepared injectable hydrogels with enhanced lubrication and controlled drug release for OA therapy by using a micro-control technique to prepare GelMA microspheres (Han et al., 2021a), which were combined with the DMA-MPC bionic lubrication coating successfully prepared in previous experiments to generate lubricated hydrogel microspheres (GelMA@DMA-MPC). The GelMA microspheres contribute to the lubrication performance, while the amphoteric choline phosphate groups in the copolymer further synergistically reduce the CoF. As a result, the lubrication performance of this hydrogel microsphere is very effective (μ ≈ 0.019); its encapsulation with the anti-inflammatory drug diclofenac sodium (DS) for drug release function provides a highly promising therapeutic modality for OA. Similarly, nano fat (NF) is widely used for treating various diseases due to its rich bioactive components, and its inability to be positioned during implantation limits its biofunctional application. Han et al. (2022) immobilized nano fat in microfluidically generated aldehyde-modified poly(lactic-co-glycolic acid) (PLGA) through Schiff base condensation and covalent bonding in a three-dimensional porous network (PMs@NF) porous microspheres, which improved the cartilage targeting efficiency of the nano fats, not only inhibited the progression of OA but also led to significant lubricating properties, reducing the CoF by approximately 80%, with the lowest CoF when the PMs@NF complex ratio was 1:3 (1 N: 0.1393; 2 N: 0.1390; 3 N: 0.1365; 4 N: 0.1837). This material has shown great potential in the treatment of OA.

We reviewed many high-performance hydrogels that have emerged, and these hydrogel materials are dedicated to improving the comprehensive performance mainly in terms of lubrication and mechanical properties. However, there is still a considerable gap between the performance of most bionic materials and natural cartilage. Many solutions to these problems have been proposed, such as multi-network structure hydrogels, bilayer hydrogels composed of different functional layers, and the addition of pro-lubricant molecules, etc. These methods have certain breakthroughs and application prospects, and further *in vivo* experiments are needed to have them become clinically applicable products.

#### 4.1.3 Bionic cartilage material hydrogel coating

When developing bionic materials for articular cartilage replacement, in addition to meeting the requirements of mechanical properties, it is also necessary that such materials can be tightly bonded to the bone substrate. Many studies are currently focused on improving the mechanical properties and ignoring the bonding issues, which can lead to severe consequences: inflammation and other traumas, which significantly limit the application of hydrogels in artificial joint cartilage.

Mredha et al. (2017) developed a novel collagen fiber-based tough hydrogel by using fish bladder collagen (SBC), in which SBC was used as the first mesh and poly(N, N′-Diethylacrylamide) (PDMAAm) as the second cross-linked network. Due to its anisotropic swelling behavior, this dual-network hydrogel exhibited excellent mechanical properties comparable to natural cartilage. Furthermore, after covering this DN gel with a hydroxyapatite (HAp) coating, the HAp/SBC/PDMAAm gel was firmly bonded to the bone after 4 weeks.

Ma et al. (2015b) obtained an anodic aluminum oxide (AAO) and acrylic acid (AA) composite to obtain an ordered arrangement of hydrogel fiber-alumina composite, a material with nanopores that can confine the hydrogel fibers to its interior. At the same time, the water loss and contraction of the surface gel pull out the hydrogel fibers partially to form a fiber array. Due to the soft and hard composite of this fiber array interface, the mechanical deformation of the interface during shear is reduced, thus exhibiting excellent lubrication properties.

Chen et al. (2015) formed reactive groups on the surface of ultrahigh-molecular-weight polyethylene (UHMWPE) by oxidation of UHMWPE with dichromate. Then the hydroxyl groups in the PVA hydrogel molecules were catalyzed by a catalyst to graft the reactive groups on the UHMWPE surface chemically. Then they tested the effects of temperature, grafting time, catalyst amount, and grafting solution concentration on the grafting effect were explored. The experimental results showed that after chemical grafting, the shear strength of artificial joints could reach 1 MPa (9.9 atm, 1 MPa), and the contact angle of UHMWPE was reduced from 104° to 39°, effectively improving the surface lubrication properties.

Yuk et al. (2016) cross-linked polyacrylamide (PAAM) and poly(ethylene glycol) while acrylate (PEGDA) to various surface-modified solids, respectively, to achieve the hydrogel with different substrate surfaces. The interfacial toughness between such hydrogels and substrates was even better than that between tendon-bone and cartilage-bone interfaces, reaching more than 1,000 J/m^2^.

Han et al. (2021b) successfully synthesized a self-adhesive copolymer material (DMA-MPC) by free radical polymerization using dopamine methacrylamide (DMA) and MPC as raw materials, with dopamine acting as an anchoring and embedding agent to promote the gradual assembly of multilayer coatings, and attached it to Ti6Al4V in the form of a coating. The experimental results showed that the CoF of Ti6Al4V with DMA-MPC polymer coating was reduced to different degrees (with the most considerable reduction from 0.136 to 0.025 for DMA/MPC = 1:4). At the same time, this coating can prevent or delay the adsorption of proteins or bacteria to achieve the antibacterial effect. This self-adhesive lubricating coating has good lubricating and antibacterial properties, which has a promising application in bionic cartilage materials.

#### 4.1.4 Hydrogel-based delivery system achieved by stem cell components

**FIGURE 4:**
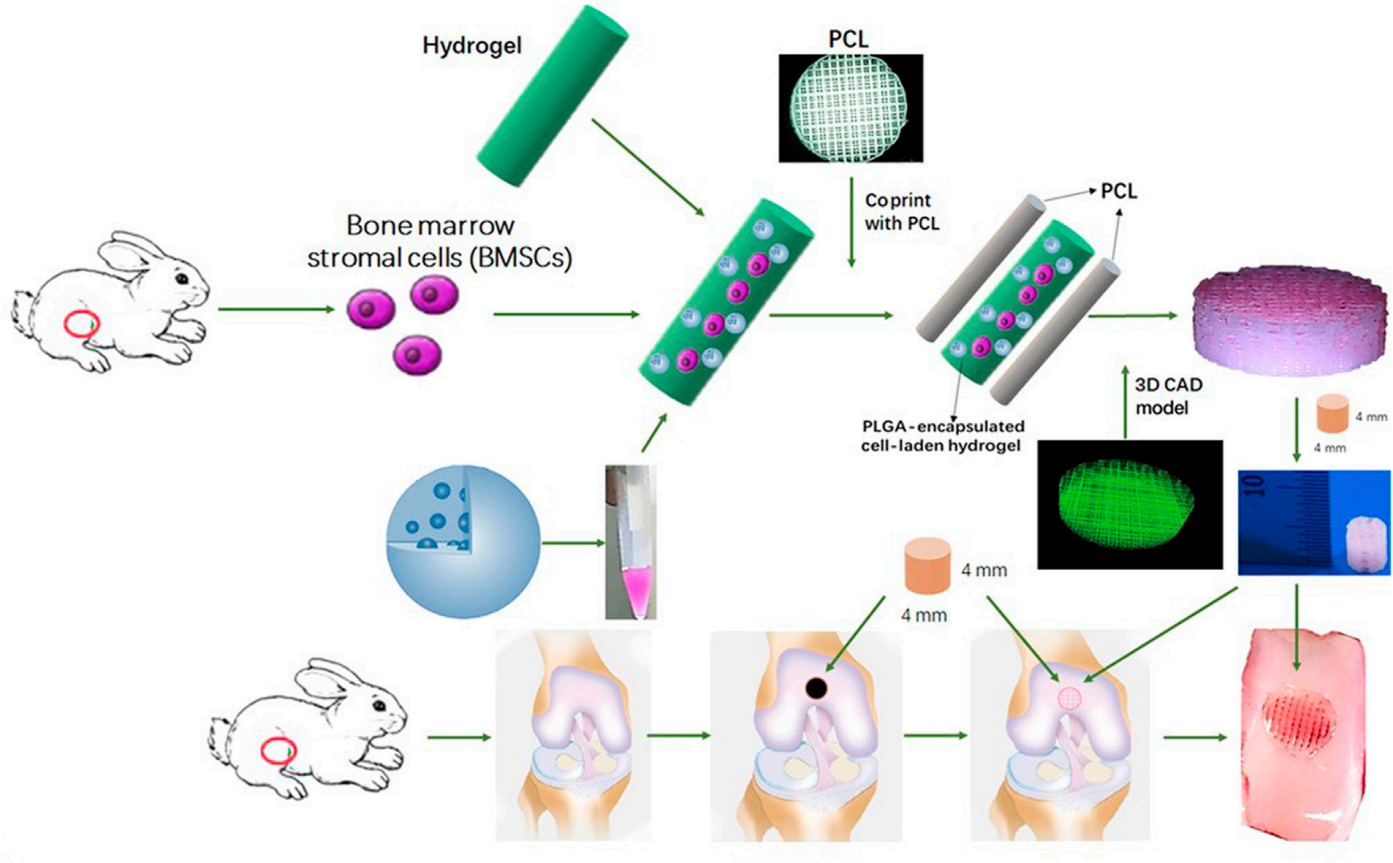
Schematic Illustration of the study design with 3D bioprinted dual-factor releasing and gradient-structured MSC-laden constructs for articular cartilage regeneration in rabbits. Schematic diagram of construction of the anisotropic cartilage scaffold and study design (Sun et al., 2020). Copyright 2020, Science Adv.

Mesenchymal stem cells (MSC) are a common cell type used in regenerative medicine. MSC possess excellent homing ability, proliferation and multi-lineage differentiation potential (Zhang et al., 2018). According to previous clinical trials, MSC are not attacked by the human immune system due to the lack of MHC II antigen, and Occhetta et al. (2018) found that the use of MSC transplants followed by stimulation of their targeted differentiation into chondrocytes contributed to cartilage repair. It has been shown that scaffolds with small pore sizes contribute to the proliferation and differentiation of MSC. Therefore, Sun et al. (2020) used 3D printing to combine hydrogel and poly(ε-caprolactone) (PCL) to construct a PCL-hydrogel composite. This composite delivered MSC and induction factors and was implanted into a rabbit joint with a total cartilage defect. It was observed that this composite provided MSC with a microenvironment conducive to differentiation into chondrocytes and exhibited better cartilage repair (Sun et al., 2020) (Figure 4).

In previous studies, several miRNAs (mir-29b-5p, e.g.) targeting OA-related genes were found to have clinical value for the treatment of OA (Vicente et al., 2016). Using an injectable hydrogel with good lubricating properties to deliver mir-29b-5p, Zhu et al. (2022) effectively inhibited abnormal metabolism of the cartilage matrix as well as endogenous recruitment of synovial stem cells, which effectively promoted cartilage repair and chondrocyte regeneration.

### 4.2 Bio-inspired bio-lubricants

In the previously mentioned lubrication model, molecules such as HA, lubricin, and phospholipids present in the organism play an important role in articular cartilage lubrication. Therefore, more synthetic bio-lubricants have been investigated around these molecules for reducing cartilage friction. The typical bio-inspired cartilage lubrication materials mentioned in section 4 are listed in Table 1. In 2018, Han et al. (2018) developed an ultra-smooth polyelectrolyte coating *via* the co-deposition of synthesized poly(dopamine methacrylamide-co-2-methacryloyloxyethyl phosphorylcholine) (P(DMA-co-MPC)) and dopamine (DA) in a mild condition. The coating is simple to manufacture and has excellent lubricating properties (Han et al., 2018). In the same year, Morgese et al. (2018) designed tissue-reactive graft copolymers, which has excellent lubricating properties and protects cartilage from enzymatic degradation. In the 2021, Wan et al. (2021) proposed a nanostructured film based on hydrophilic polysaccharide hyaluronic acid conjugated with dopamine (HADN) and zwitterionic reduced glutathione, this special composite coating (HADN-Glu) has demonstrated better lubricating properties in vitro experiments.

**TABLE 1:** Typical bio-inspired cartilage lubrication materials.

Bio-inspired lubrication materials	Manufacturing methods	References
DN hydrogels	Combining a relatively rigid polyelectrolyte as the primary network with a neutral polymer with low cross-linkage as the secondary network	Lin and Klein (2021)
Triple-network hydrogels	Adding a third, negatively charged, weakly cross-linked network to the structure of the DN hydrogels	Kaneko et al. (2010)
PMPC triple network hydrogel	Incorporating poly(2-methacryloyloxyethyl phosphorylcholine) (PMPC) with DN hydrogels	Milner et al. (2018)
β-TCP/PVA bilayered hydrogel	Blending polyvinyl alcohol (PVA) and β-tricalcium phosphate (β-TCP)	Yao et al. (2017)
Double networks of covalent crosslinks hydrogel	Combining ionically crosslinked alginate with covalently crosslinked polyacrylamide	Sun et al. (2012)
poly(hydroxyethylmethacrylate) (pHEMA) hydrogel	Mixing a low concentration of PC lipids with the desired monomer solution, then polymerizing and cross-linking to form the hydrogel	Lin et al. (2020)
Semi-interpenetrating network composite gel (CG)	Incorporating short chain chitosan (CS) into a covalent tetra-armed poly(ethylene glycol) network	Zhang et al. (2022)
Bilayered Composite Hydrogel	Robustly entangling thick hydrophilic polyelectrolyte brushes into the subsurface of a stiff hydrogel substrate	Rong et al. (2020)
Cartilage-inspired layered hydrogel material	Employing an alkali-induced network dissociation strategy	Qu et al. (2020)
Photocrosslinked GelMA/PAMAM-MA hydrogel	Incorporating methacrylic anhydride-modified poly(amidoamine) (PAMAM-MA) into the photocrosslinked gelatin methacrylate (GelMA) hydroge	Liu et al. (2022)
Chitosan-g-PMPC copolymers	Combines natural polysaccharide (chitosan) with zwitterionic poly[2-(methacryloyloxy) ethyl phosphorylcholine] (PMPC)	Yue et al. (2022)
Composite-LP	Grafting a thick hydrophilic polyelectrolyte brush layer onto the subsurface of a three-dimensional manufactured elastomer scaffold-hydrogel composite architecture	Zhao et al. (2022)
Hydrogel-elastomer hybrid surface	The hydrogel interpenetrates into the polymer elastomer substrate as a transitional and bonding zone	Huang et al. (2021)
Rapamycin-liposome-incorporating hyaluronic acid-based HMs (RAPA@Lipo@HMs)	Using microfluidic technology and photopolymerization processes	Lei et al. (2022a)
Shear-responsive boundary-lubricated drug-loaded hydrogel	Incorporating celecoxib (CLX)-loaded liposomes within dynamic covalent bond-based hyaluronic acid (HA) hydrogels (CLX@Lipo@HA-gel)	Lei et al. (2022b)
MGS@DMA-SBMA	One-step biomimetic grafting approach	Yang et al. (2020)
A collagen fibril-based tough hydrogels, SBC/PDMAAm	Composed of SBC fibril and PDMAAm	Mredha et al. (2017)
Crosslinked polymethylacrylic acid hydrogels	Based on a soft/hard combination strategy	Chen et al. (2015)
DMA-MPC copolymer	Composed of DMA and MPC	Han et al. (2021b)
Ultra-smooth polyelectrolyte coating	Co-deposition of (P(DMA-co-MPC)) and dopamine	Han et al. (2018)
Tissue-reactive graft copolymers	Synthesizeing the cyclic macromolecules	Morgese et al. (2018)
A special composite coating (HADN-Glu)	Synthesized by carbodiimide chemistry between hyaluronic acid and dopamine and deposited on PCU surface under mild oxidative conditions	Wan et al. (2021)

## 5 Conclusion and prospects

In this review, we briefly described the theories of articular cartilage lubrication mechanism and the progress of bionic cartilage materials. Articular cartilage plays an essential and irreplaceable role in joint activities, and its wear and tear due to trauma and aging can lead to severe consequences such as osteoarthritis. At the same time, articular cartilage is characterized by poor nutrient supply and metabolic circulation, resulting in poor self-healing ability. Therefore, intervention at an early stage of the disease, such as restoring the lubricity of the articular cartilage or replacing it with artificial cartilage, is the primary way to maintain normal joint function. In the past few decades, researches of cartilage lubrication has progressed rapidly and a variety of theories have been elucidated, leading the new biomimetic cartilage materials development.

Articular cartilage lubrication is essential for normal joint movement and studying the mechanisms of efficient cartilage lubrication at the molecular level helps to better explain its complex mechanisms. More and more studies are able to restore the very low friction between joints close to the human body in the laboratory, which also offers the possibility of clinical treatment of joint diseases such as OA. With regard to the advancement of bionic cartilage materials, many promising cartilage replacement materials are emerging. Hydrogels have extremely important applications in the field of bionic articular cartilage due to their lubricating properties similar to those of articular cartilage. In addition to the hydrogel, more synthetic bio-lubricants inspired by endogenous molecules associated with lubrication have been developed. We believe that biomimetic lubrication materials with more natural components from human micro environment will be developed to provide new solutions for the prevention and treatment of joint diseases on the premise of ensuring biological safety and close to physiological lubrication mechanism.
